# A combination of LMO2 negative and CD38 positive is useful for the diagnosis of Burkitt lymphoma

**DOI:** 10.1186/s13000-019-0876-3

**Published:** 2019-09-04

**Authors:** Yifei Liu, Tingting Bian, Yanlin Zhang, Yuanyuan Zheng, Jianguo Zhang, Xiaoge Zhou, Jianlan Xie

**Affiliations:** 1 Department of Pathology, Affiliated Hospital of Nantong University, Nantong, 226001 People’s Republic of China; 2 Department of Pathology, Beijing Friendship Hospital, Capital Medical University, Beijing, 100000 People’s Republic of China

**Keywords:** LMO2, CD38, Burkitt lymphoma, Immunohistochemistry

## Abstract

**Background:**

To evaluate the clinical utility of LIM Domain Only 2 (LMO2) negative and CD38 positive in diagnosis of Burkitt lymphoma (BL).

**Methods:**

LMO2 and CD38 expression determined by immunohistochemistry in 75 BL, 12 High-grade B-cell lymphoma, NOS (HGBL,NOS) and 3 Burkitt-like lymphomas with the 11q aberration.

**Results:**

The sensitivity and specificity of LMO2 negative for detecting BL were 98.67 and 100%, respectively; those of CD38 positive were 98.67 and 66.67%, respectively. The sensitivity and specificity of a combination of both for detecting BL were 97.33 and 100%, respectively. In our study, the combined LMO2 negative and CD38 positive results had a higher area under the curve than either LMO2 negative or CD38 positive alone.

**Conclusions:**

A combination of LMO2 negative and CD38 positive is useful for the diagnosis of Burkitt lymphoma.

## Background

Burkitt lymphoma (BL) is one of the most studied human malignant tumors that originates in the B cells. Although it is relatively simple to diagnosis BL in children, it is a challenge to identify reliable subtypes of aggressive B-cell lymphoma in adults [1, 2]. It is crucial to distinguish BL from other lymphomas because of its rapid progress and the planned improvements in treatment for adult aggressive B-cell lymphomas [1–4].

BL is a highly aggressive B-cell lymphoma with unique morphologic, immunophenotypic, and molecular features [5]. BL tumor cells are monomorphic, composed of medium-sized cells with round nuclei, multiple deeply stained nucleoli, and basophilic cytoplasm. The cell proliferation rate as well as the apoptotic rate are extremely high. Approximately 100% of the cells are Ki-67 positive (MIB-1 positive) and display the “starry sky” pattern. BL has a typical immunophenotype-strong immunoglobulin (Ig) expression and generally expresses markers of B cell-associated antigens (CD19, CD20, CD22, and CD79a) and a germinal center (CD10). It does not express BCL-2 [6]. In nearly all studies, BL was associated with one of three chromosomal translocations on the *c-MYC* oncogene locus (8q24) and the *Ig* gene on the long arm of chromosome 14*,* also the immunoglobulin light chain genes on chromosomes 2 and 22 [7–9].

High-grade B-cell lymphoma, NOS includes blastoid-appearing large B-cell lymphomas and cases lacking *MYC* and *BCL2* or *BCL6* translocations. *HGBL*, with *MYC* and *BCL2* and/or *BCL6* and *HGBL, NOS* replaces the 2008 category of B-cell lymphoma, unclassifiable, with features intermediate between DLBCL and Burkitt lymphoma (BCLU) [5]. Most morphologic features are intermediate between those of DLBCL and BL, with a high proliferative index and starry sky pattern, and the immunophenotype is consistent with that of BL.

Burkitt-like lymphoma with an 11q aberration has morphologic and immunophenotypic features similar to those of BL, but lacks *MYC* rearrangement and has the typical 11q aberration, which appears as a partial amplification and partial deletion in the region at the same time [10]. The tumor is rare, accounting for only 3% of BL, is more common in children and young people and more in males than females, and is more likely to involve lymph nodes than BL [11].

The above lymphomas are difficult to distinguish from their histological morphologies and existing routine immunophenotypes. Our study hopes to discover new immunohistochemical markers and analyze their expressions in these lymphomas so as to better diagnosis of BL.

LMO2 is a transcription factor that plays an important role in embryonic development and angiogenesis. Studies have shown that many tumors have LMO2 expression and that it is associated with the prognosis for patients with certain tumors, such as glioblastoma and pancreatic cancer [12, 13]. In the lymphatic and hematopoietic system, in addition to expression in the normal lymphoid germinal center, LMO2 is expressed in germinal center-derived lymphomas, acute B-lymphoblastic leukemia, and acute myeloid leukemia (AML) [14]. Recent studies have found that LMO2 protein expression is downregulated or negative in BL with abnormal *MYC* [2]. CD38 is a type II transmembrane glycoprotein that has several complex and unique biological characteristics and functions. It is widely expressed in both hematopoietic and non-hematopoietic cells, including bone marrow precursor cells, germinal center B-cells, plasma cells, prostate epithelial cells, skeletal muscle, and other tissues, and in activated T cells, B cells, monocytes, NK cells, and islet cells [15]. CD38 is strongly expressed in both plasma cells and plasma cell tumors. It is also present in acute lymphoblastic leukemia, AML, chronic lymphocytic leukemia, and non-Hodgkin lymphoma (NHL) [16, 17]; however, no in-depth studies have been conducted to verify the positive expression of CD38 in BL.

Our study analyzed the expression of LMO2 and CD38 proteins in BL, HGBL,NOS and Burkitt-like lymphomas with the 11q aberration and hypothesized that the combination of LMO2-negative and CD38-positive expressions can be used to diagnose auxiliary BL. To test this hypothesis, we analyzed the specificity and sensitivity of LMO2-negative, CD38-positive, and the combination of both expressions, as well as their diagnostic efficiency in BL.

## Materials and methods

### Case selection

From May 2015 to March 2018, we compiled 75 cases of BL, 12 cases of HGBL, NOS and 3 cases of Burkitt-like lymphoma with the 11q aberration from the Department of Pathology in Beijing Friendship Hospital, Capital Medical University, China. All cases were classified according to the diagnostic criteria of the 2016 revision of the World Health Organization (WHO) Classification of Tumours of Haematopoietic and Lymphoid Tissues. None of the patients received any treatment and all had complete pathological data. The study was retrospectively performed and was approved by the Ethics Committee of Beijing Friendship Hospital, Capital Medical University (2018-P2–130-01).

### Immunohistochemistry

All samples were fixed with 3.7% neutral formaldehyde, followed by routine paraffin section and hematoxylin and eosin staining. Proteins CD38 (clone 38CO3), CD10 (clone MX002), BCL-6 (clone LN22), BCL-2 (clone SP66), MUM1 (clone MUM1p), c-Myc (clone Y69), Ki67 (clone MIB-1), their reagents, and their primary antibodies were purchased from the Fuzhou Maixin Biotechnologies Development Company (Maixin, Fuzhou, China).

The conditions and the evaluation of all these antibodies were the same as those previously described and were assessed following the recommended guidelines for their interpretation by the Luneburg Lymphoma Biomarker Consortium; appropriate internal controls were used in the evaluation of the immunostains [18, 19]. c-Myc, CD38, and Ki67 immunostaining were also semiquantitatively evaluated, and the cutoff rates for positive results were 80, 80, and 90% [2], respectively.

LMO2 was studied using clone 1A9–1 (Ventana,Roche, Tucson, AZ), which was detected using the ultraView Universal DAB Detection Kit (Ventana Medical Systems, Tucson, AZ, USA) in the BenchMark XT automated immunostainer (Ventana). LMO2 immunostaining was evaluated following the cutoff criteria by Natkunam et al. [14], and in which staining of > 30% of the lymphoma cells was designated as positive for LMO2.

Brownish-yellow nuclear particles were observed in cells staining positive for LMO2 and c-Myc. Cells were defined as CD38 positive when the cell membrane stained brownish yellow.

### Detection using fluorescence in situ hybridization

FISH was conducted using the ATM dual color probe (LBP Medicine Science and Technology Co., Ltd., Guangzhou, China). *ATM* (11q22.3) was marked in red, and the CEP11 (11p11–q11) chromosomal probe was labeled in green. In addition, the *MYC* break apart probe (Beijing GPmedical Technology Co., Ltd.) was used to detect *MYC* status. The specific operations were conducted according to the manufacturers’ instructions.

### Statistical analyses

Staining sensitivity and specificity for LMO2 and CD38 with 95% exact binomial confidence intervals (95%CIs) were calculated. Our immunostaining criteria for diagnosing BL were positive staining for CD38 and negative staining for LMO2.

Data were compared using the χ2 test, unpaired t-tests, or nonparametric tests, when necessary. *P* < .05 was considered statistically significant for all tests. The differences between rates were tested using χ2 or Fisher’s exact tests, when appropriate.

Logistic regression was used to model BL as a function of immunostaining. The corresponding receiver operating characteristic (ROC) curves were plotted for different combinations of immunostains, and the areas under these correlated ROC curves (AUCs) were compared using the nonparametric approach of DeLong et al. and integrated discrimination improvement index (IDI) [20, 21]. All analyses were performed using SPSS v 21.0 (IBM Corp., Armonk, NY, USA) and MedCalc v 9.2.1.0 (https://www.medcalc.org/).

## Results

### Clinicopathological and immunohistochemical features

The clinicopathological features and the expression of immunohistochemical markers in 75 cases of BL, 12 cases of HGBL,NOS and 3 cases of Burkitt-like lymphoma with the 11q aberration are shown in Table 1.

**Table 1:** The clinicopathologic features and the expression of immunohistochemical markers in Burkitt lymphoma, High-grade B-cell lymphoma, not otherwise specified and Burkitt-like lymphoma with 11q aberration

Characteristic	n (%)		
	BL *N* = 75	HGBL, NOS *N* = 12	11q *N* = 3
Median age (range)	10(2–69)	31(1–67)	15(10–22)
Male	62 (82.67)	7 (58.33)	1 (33.33)
Intranodal sites	27 (36.00)	4 (33.33)	1 (33.33)
LMO-2-	74 (98.67)	0 (0.00)	3 (100.00)
CD38+	74 (98.67)	4 (33.33)	3 (100.00)
c-Myc+	67 (89.33)	5 (41.67)	2 (66.67)
Ki67+	64 (85.33)	8 (66.67)	2 (66.67)
BCL-2-	67 (89.33)	10 (83.33)	2 (66.67)
MUM1+	42 (56.00)	4 (33.33)	2 (66.67)
BCL6+	73 (97.33)	11 (91.67)	3 (100.00)
CD10+	73 (97.33)	11 (91.67)	3 (100.00)

*LMO2* LIM Domain Only 2, *BL* Burkitt lymphoma, *HGBL, NOS* High-grade B-cell lymphoma, NOS

**Fig. 1:**
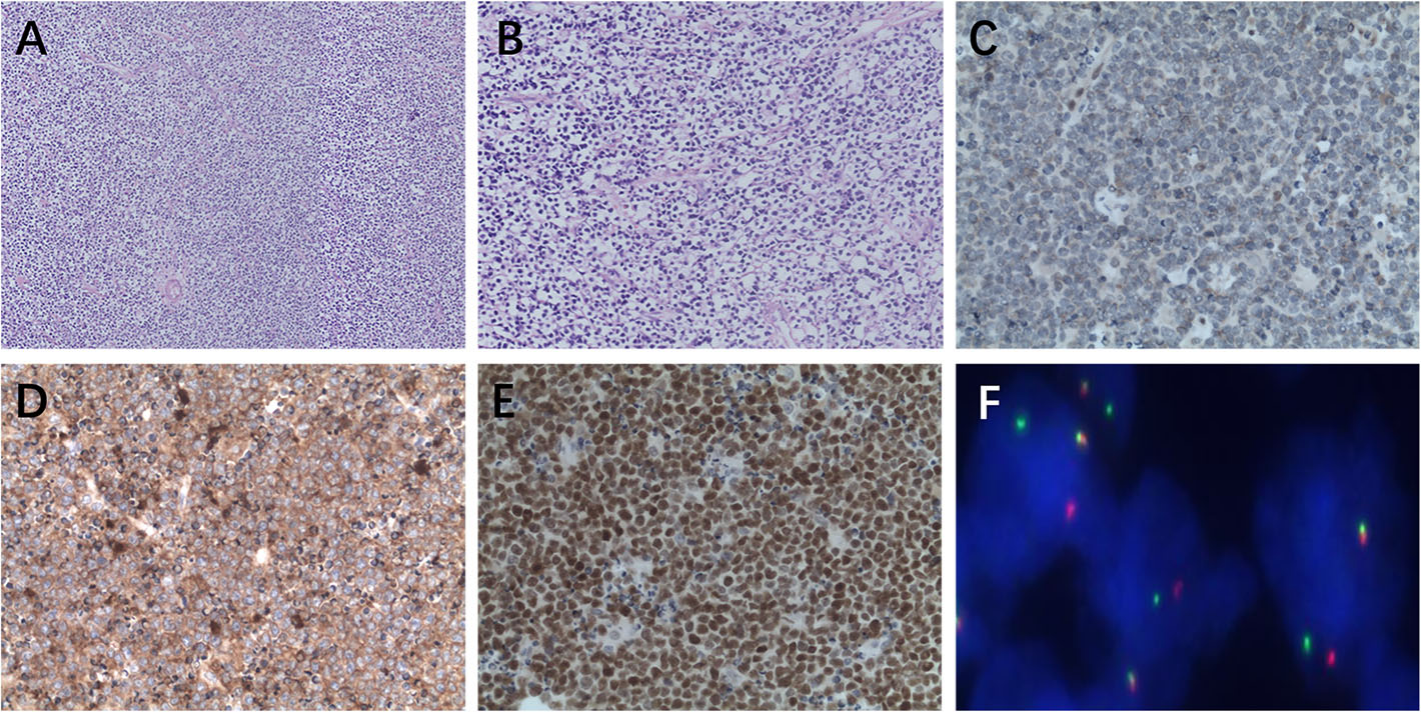
Burkitt lymphoma showing low- and high-magnification (**a**, **b**) not LMO2 expression (**c**) but CD38 and c-Myc expression (**d**, **e**); BL with *MYC* rearranged (**f**)

Of the 75 cases of BL, 62 were males, and patient ages ranged from 2 to 69 years with a median age of 10 years. Of the 75 BL cases, 27 involved lymph nodes and 48 were extranodal. Morphologically, the tumors consisted of sheets of a monotonous population of tumor cells with diffuse infiltration. They were closely packed, medium sized, had small or medium amounts of cytoplasm, were lightly stained, contained a round nucleus, exhibited a coarse chromatin pattern, and contained two to four small nucleoli within each nucleus. A large number of nuclear divisions were observed within the tumor, and a large number of dead neoplastic cells that were swallowed by macrophages to form a “starry sky” phenomenon (Fig. 1a, b) were also observed. Of the 75 cases of BL, 74 (98.67%) were negative for LMO2 and positive for CD38. The expression rates of CD10+, BCL-6+, BCL-2-, MUM-1+, c-Myc (80%+), and Ki67 (95%+) were 73/75 (97.33%), 73/75 (97.33%), 67/75 (89.33%), 42/75 (56%), 67/75 (89.33%), and 64/75 (85.33%), respectively. The BL tumor cells were generally negative for LMO2, but were strongly and diffusely cell membrane positive for CD38, and ≥ 80% of tumor cells were strongly nuclear positive for c-Myc (Fig. 1c-e).

**Fig. 2:**
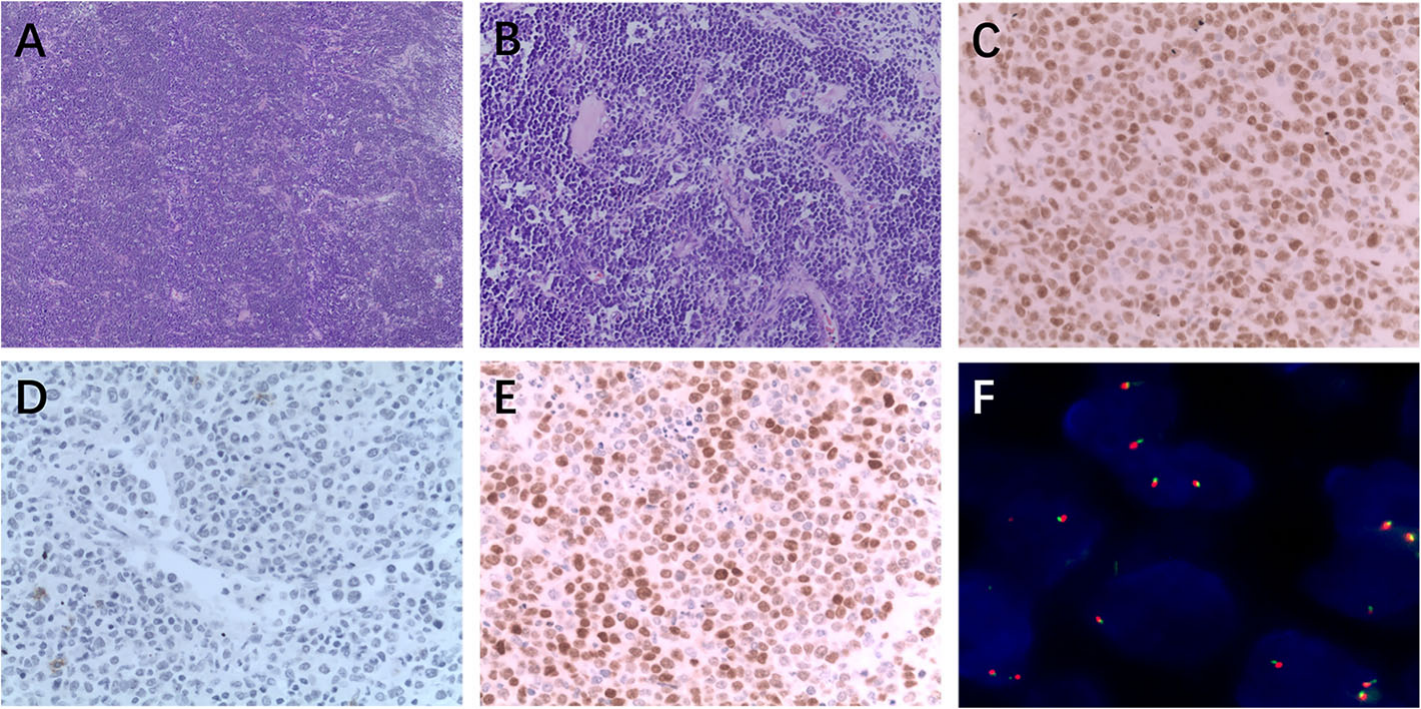
High-grade B-cell lymphoma, not otherwise specified (HGBL, NOS) showing low- and high-magnification (**a**, **b**) LMO2 expression(**c**) but not CD38 expression(**d**) and c-Myc expression(**e**); HGBL, NOSwith *MYC* nonrearranged(**f**)

Seven of the 12 patients with HGBL,NOS were males. Patient ages ranged from 1 to 67 years with a median age of 31 years. Four cases involved lymph nodes and eight were extranodal. Morphologically, these gray areas or borderline cases were characterized by medium-size cells that were similar to those in BL and mixed with some of the large cells typically seen in DLBCL (Fig. 2a, b). All 12 cases showed 100% (12/12) expression of LMO2. Four (4/12) cases (33.3%) were positive for CD38. The expression rates of CD10+, BCL-6+, BCL-2-, MUM-1+, c-Myc (80%+), and Ki67 (95%+) were 11/12 (91.67%), 11/12 (91.67%), 10/12 (83.33%), 4/12 (33.33%), 5/12 (41.67%), and 8/12 (66.67%), respectively. In HGBL, LMO2 was found in moderate intensity in the nucleus, CD38 was not expressed or was weakly expressed in the tumor cells, and c-Myc was detected in some tumor cell nuclei (Fig. 2c-e).

**Fig. 3:**
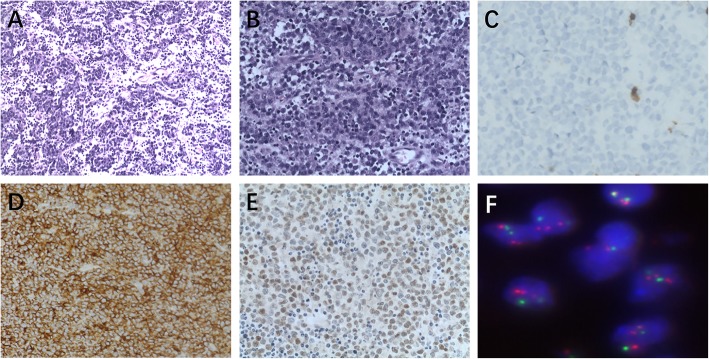
Burkitt-like lymphomas with the 11q aberration showing low- and high-magnification (**a**, **b**) not LMO2 expression (**c**) but CD38 and c-Myc expression (**d**, **e**); Burkitt-like lymphomas with the 11q aberration showed that *ATM* (11q22.3) was amplified (**f**)

One of the three patients with Burkitt-like lymphoma with the 11q aberration was male. The age of the three patients was 10, 15 and 22 years respectively. One case involved lymph nodes and two were extranodal. Morphologically, the tumors were very similar to those of BL, appearing as diffusely growing, medium-sized lymphocytes with uniform cells. There were multiple deviated small nucleoli scattered within the tingible body macrophages to form a starry sky phenomenon (Fig. 3a, b). All three cases were LMO2 negative and CD38 positive (100%). The expression rates of CD10+, BCL-6+, BCL-2-, MUM-1+, c-Myc (80%+), and Ki67 (95%+) were 3/3 (100%), 3/3 (100%), 2/3 (66.67%), 2/3 (66.67%), 2/3 (66.67%), and 2/3 (66.67%), respectively. In Burkitt-like lymphoma with the 11q aberration, the expression patterns of LMO2, CD38, and c-Myc in the tumor cells were similar to those in BL tumor cells (Fig. 3c-e).

### FISH detection results

In BL, *c-MYC* translocation showed one red signal, one green signal, and one fused yellow signal in the nucleus (Fig.1f). In HGBL,NOS showed *MYC* nonrearranged (Fig. 2f). In the Burkitt-like lymphoma with the 11q aberration, the *MYC* gene break apart FISH probe did not detect breakpoints in *MYC*. When the ATM dual color probe was used, *ATM* (11q22.3) was marked red and the CEP11 (11p11-q11) chromosomal probe was marked green. The results showed that *ATM* was amplified (three red, two green) (Fig. 3f).

### Statistical analyses of immunohistochemical expression in BL and HGBL, NOS

There were significant differences in the expression of the three immunophenotypes LMO2 negative, CD38 positive, and c-Myc (80%+) in the 75 cases of BL and 12 cases of HGBL, NOS(*P* < .01) (Table 2).

**Table 2:** The statistical analysis of immunohistochemical expression in Burkitt lymphoma and High-grade B-cell lymphoma, not otherwise specified

Characteristic	n (%)		P
	BL *N* = 75	HGBL, NOS *N* = 12	
Intranodal sites	27 (36.00)	4 (33.33)	0.858
LMO-2-	74 (98.67)	0 (0.00)	< 0.01
CD38+	74 (98.67)	4 (33.33)	< 0.01
c-Myc+	67 (89.33)	5 (41.67)	< 0.01
Ki67+	64 (85.33)	8 (66.67)	0.112
BCL-2-	67 (89.33)	10 (83.33)	0.545
MUM1+	42 (56.00)	4 (33.33)	0.144
BCL6+	73 (97.33)	11 (91.67)	0.318
CD10+	73 (97.33)	11 (91.67)	0.318

*LMO2* LIM Domain Only 2, *BL* Burkitt lymphoma, *HGBL, NOS* High-grade B-cell lymphoma, NOS

### Sensitivity and specificity of immunostaining combinations

The sensitivities (95%CI) of tissues staining LMO2 negative, CD38 positive, and a combination of LMO2 negative and CD38 positive were 98.67, 98.67, and 97.33%, respectively. The corresponding specificities (95%CI) were 100, 66.67, and 100%, respectively (Table 3). The ROC curves for the immunohistochemistry markers were analyzed by logistic regression. The AUC (95%CI) for tissues staining LMO2 negative, CD38 positive, and a combination of LMO2 negative and CD38 positive were 0.993, 0.827, and 0.998, respectively (Table 3).

**Table 3:** Sensitivity and specificity of immunostaining combinations

	Sensitivity (95% CI)	Specificity (95% CI)	AUC (95% CI)
LMO2-	98.67 (92.8–100.0)	100 (73.5–100.1)	0.993 (0.946–1.000)
CD38+	98.67 (92.8–100.0)	66.67 (34.9–90.1)	0.827 (0.731–0.899)
LMO2- & CD38+	97.33 (90.7–99.7)	100 (73.5–100.9)	0.998 (0.954–1.000)

*LMO2* LIM Domain Only 2, *CI* confidence interval, *AUC* area under the curve

### Comparison of the diagnostic efficacy between combination of LMO2 negative and CD38 positive and single index

A significant difference existed between ROC curves for tissues staining LMO2 negative and CD38 positive compared with those staining CD38 positive (*P* = .015); however, there was no significant difference observed between those staining LMO2 negative and those staining both LMO2 negative and CD38 positive (*P* = .328) (Table 4). The same results can be obtained by integrated discrimination improvement index analysis (Table 4).

**Table 4:** Comparison of the diagnostic efficacy between combination of LMO2(−) and CD38(+) and single index

	AUC		IDI		
	ΔAUC	*P* value	ΔIDI	Z	P value
LMO2-	0.005	0.328	0.012	0.131	0.896
CD38+	0.171	0.015	0.375	2.243	0.025

*LMO2* LIM Domain Only 2, *AUC* area under the curve, *IDI* integrated discrimination improvement index

## Discussion

BL is a highly aggressive B-cell NHL characterized by the translocation and dysregulation of *c-MYC* on chromosome 8 [2]. Researchers have questioned whether *c-MYC* rearrangement is a necessary condition for the diagnosis of BL and have found that ≤5% of the tumors with typical BL characteristics do not have *c-MYC* rearrangement [1, 22]. Some researchers have speculated that these cases might have molecular pathogeneses other than the *MYC* activation mechanism, which is the BL’s iconic pathogenesis. Recently, many studies have reported cases with clinical, morphologic, immunophenotypic, or gene expression characteristics consistent with BL, but lacked FISH-detected positive *MYC* rearrangement. Additional studies have found that there were 11q aberrations in MYC-negative cases [10, 11, 23]; therefore, the 2016 revision of *WHO Classification of Tumours of Haematopoietic and Lymphoid Tissues* proposed a new temporary type of lymphoma-Burkitt-like lymphoma with the 11q aberration [5].

BL, HGBL,NOS and Burkitt-like lymphoma with the 11q aberration can be diffusely infiltrated by large, medium-sized lymphocytes, no obvious nodule formation, monotonous and consistent cells, and a starry sky pattern. In addition to the expression of B-cell markers, all tumors showed mostly the expression of CD10 positive, BCL-6 positive, and BCL-2 negative in the immunophenotype; therefore, these types of tumors cannot be fully identified using only their morphology and the immunophenotype.

Previous studies have found that LMO2 has high sensitivity and specificity of expression in normal germinal center B cells and germinal center B cell-derived lymphomas. LMO2 was also expressed in myeloid and erythroid progenitor cells, megakaryocytes, lymphocytes, and acute myeloid leukemia. It was rarely expressed in mature T cells, natural killer (NK) cells, or plasma cell tumors. In addition, with the exception of endothelial cells, it did not express in non-lymphoid hematopoietic tissue. In DLBCL, the expression profile of LMO2 was similar to that of other germinal center-related proteins (HGAL, BCL6, and CD10), but was different from nongerminal center proteins (MUM1/IRF4 and BCL2) [14]. Recent studies have found that LMO2 might be a useful indicator for identifying *MYC* translocation and might also help identify BL [24].

We observed that the deletion of LMO2 expression might be particularly helpful in diagnosing BL. In this series, we found 74 of the 75 BL cases studied were negative for LMO2 using a cutoff of 30%. This was consistent with the data obtained using the GEP study, which indicated that the expression level of LMO2 was lower in BL [1, 2]. Only three studies analyzed the expression of the LMO2 protein in a small number of BL cases. Natkunam and colleagues and Agostinelli and colleagues defined two different cloned LMO2 proteins and evaluated the specificity and effectiveness of their antibodies. In these two studies, the expression rate of LMO2 in BL was 5/10 (50.0%) and 13/32 (41.0%), respectively, and 1/3 (33.3%) in the BL cell line (Ramos cell line). A third study comprised five cases of BL, and LMO2 was expressed in only one case (20%) [14, 25, 26]. Previous studies have also found that the presence of LMO2 protein can distinguish BL from DLBCL [25], because it was more commonly expressed in the latter [26]. In our study, we found that LMO2 protein was 100% positively expressed in HGBL; however, it was only expressed in one of Seventy-five Burkitt lymphomas. There was a statistically significant difference in the negative expression of LMO2 protein between BL and HGBL,NOS(*P* < .01). The sensitivity and specificity of the negative expression of LMO2 protein were 98.67 and 100%, respectively, and AUC of diagnostic efficiency was 0.993; therefore, we preliminarily concluded that LMO2 deletion might play a role in BL identification. None of the previous studies found a correlation between LMO2 and *MYC* rearrangements; however, a recent study not only found low expression of LMO2 in BL, but also 100% detected *MYC* rearrangement. This study suggested that the loss of LMO2 might be a good predictor of the presence of *MYC* [24]. In *MYC* rearrangement in BL, the exact mechanism that leads to LMO2 downregulation was not clear; however, Natkunam et al. [14] found that LMO2 protein is highly expressed at the mRNA level in the Ramos cell line, whereas the expression was indeed low at the immunohistochemical protein level. This suggested that LMO2 might be regulated at the posttranscriptional level in BL. These findings suggested that LMO2 protein can be used as an alternative marker for detecting *MYC* translocation in BL and might have application value in the differential diagnosis of other high-grade lymphomas.

CD38 is a transmembrane glycoprotein and in addition to marking mature plasma cells and plasma cell tumors, is a marker for germinal center B-cells [15]. Previous studies have found that CD38 is positively expressed in BL, but no in-depth studies have been conducted to verify this [27]. The expression of CD38 in HGBL,NOS and Burkitt-like lymphoma with the 11q aberration was even more limited. In our study, the positive rates of CD38 in BL, HGBL,NOS and Burkitt-like lymphoma with the 11q aberration were 98.67 (74/75), 33.3 (4/12), and 100% (3/3), respectively. There was a statistically significant difference in the positive expression rate of CD38 in BL and HGBL,NOS (*P* < .01). The sensitivity and specificity of the positive expression of CD38 protein were 98.67 and 66.67%, respectively, and AUC of diagnostic efficiency was 0.827. Previous studies have found that CD38, as LMO2, can be considered as a valuable diagnostic marker for identifying BL/DLBCL [28]. At the immunohistochemical level, it has been found that CD38 and CD44 can be used to distinguish between *MYC*-positive and *MYC*-negative lymphomas [29]. In the absence of cytogenetic analysis, it was very difficult to identify *MYC-R* in high-grade B-cell lymphomas. In practice, classical morphologic features of starry sky with medium-sized lymphocytes, typical Ki-67 hyperproliferation/CD10+/bcl-6+/bcl-2-, and recently identified CD38+/CD44−/TCL-1+ can predict a great possibility of *MYC-R* [29–40]. All of these suggest that CD38 has a specific value in the differential diagnosis of BL.

Recent studies have suggested that the expression of *MYC* protein in aggressive B-cell lymphoma can effectively predict a poor prognosis [33–40]. *MYC* protein is significantly correlated with *MYC* rearrangement, but the expression of *MYC* protein is not necessarily the result of *MYC* rearrangement [41]. In our study, the cutoff value of the positive expression of MYC protein was defined as 80% because of the differential diagnosis of BL, which was not consistent with previous studies [19, 35]. There was a significant difference in the expression of *MYC* protein in BL and HGBL,NOS (*P* < .01; Table 2) because of the defined *MYC* protein cutoff value. Because of the impact on the statistics of the defined *MYC* protein–positive cutoff value, we excluded *MYC* in subsequent statistical analyses. Finally, the combination of LMO2-negative and CD38-positive was used in the differential diagnosis of BL in our study. The sensitivity and specificity of LMO2 negative and CD38 positive were 97.33 and 100%, respectively, and AUC of diagnostic efficiency was 0.998, which was larger than AUC of those only LMO2 negative (0.993) or only CD38 positive (0.827). Further analysis found that AUC of the combination of LMO2-negative and CD38-positive was statistically different (*P* = .015) from that of CD38 positive, and there was no statistical difference (*P* = .328) in AUC of the combination of LMO2-negative and CD38-positive compared with that of LMO2 negative. The same results can be obtained by integrated discrimination improvement index analysis. The reasons for this were that first, the sample size of our study was relatively small, and in a follow-up study we will need to increase the sample size to reduce sampling error. Second, Burkitt-like lymphoma with the 11q aberration was rare; therefore, only three cases were included in our study and the expressions of LMO2 and CD38 in those cases were consistent with that in BL. We did not include these three cases in the statistical analyses shown in Table 2. Further analyses with a larger sample must be conducted to assess whether the expressions of LMO2 and CD38 in Burkitt-like lymphoma with the 11q aberration is completely identical to those in BL.

There was another limitation in our study. The best detection method for the 11q aberration is the chip technology of comparative genomic hybridization using oligonucleotide microarrays. In this study, *ATM* detected by FISH was located in 11q22. There were eight cases in the literature that reported amplification of this gene region [10, 11, 23], which was similar to the results of our study; therefore, the detection of this gene indirectly proved the 11q aberration.

## Conclusions

We believe that the assessment of LMO2 and CD38 protein expression can improve the accuracy of the pathological diagnosis of BL. At the same time, with the use of routine immune indices, such as immunohistochemical markers CD10 and BCL2, the combination of LMO2-negative and CD38-positive results can be directly applied to the routine assessment of BL in clinical practice.

## Data Availability

Is available upon request from the corresponding author.

## Abbreviations

AML: Acute myeloid leukemia
AUC: Area under the curve
BL: Burkitt lymphoma
CI: Confidence interval
HGBL, NOS: High-grade B-cell lymphoma, NOS
IDI: Integrated discrimination improvement index
NHL: Non-Hodgkin lymphoma
ROC: Receiver operating characteristic
